# Influence of Dietary Addition of Mineral Shungite and *Fucus vesiculosus* on Production Performance, Egg Quality, Nutrients Digestibility, and Immunity Status of Laying Hens

**DOI:** 10.3390/ani13203176

**Published:** 2023-10-11

**Authors:** Nikolai P. Buryakov, Anastasiya S. Zaikina, Vladimir I. Trukhachev, Maria A. Buryakova, Valentina G. Kosolapova, Ilia N. Nikonov, Ivan K. Medvedev, Mohamed M. Fathala, Dmitrii E. Aleshin

**Affiliations:** 1 Department of Feeding Animals, Institute of Animal Science and Biology, Russian State Agrarian University—Moscow Timiryazev Agricultural Academy, 127434 Moscow, Russiaazaikina@rgau-msha.ru (A.S.Z.);; 2 Department of Physiology, Ethology and Biochemistry of Animals, Institute of Animal Science and Biology, Russian State Agrarian University—Moscow Timiryazev Agricultural Academy, 127434 Moscow, Russia; 3 Department of Animal Hygiene and Poultry Breeding Named after A.K. Danilova, Faculty of Animal Technologies and Agribusiness, Moscow State Academy of Veterinary Medicine and Biotechnology—MVA Named after K.I. Skryabin, 109472 Moscow, Russia; ilnikonov@yandex.ru; 4 Animal Husbandry and Wealth Development Department, Faculty of Veterinary Medicine, Alexandria University, Alexandria 5424041, Egypt

**Keywords:** mineral shungite, dried seaweed meal, productivity indicators, nutrient utilization, blood profile, immunity status, egg quality

## Abstract

**Simple Summary:**

Searching for new feed products suitable for poultry nutrition is an important tool for improving productive and immunity status of poultry. Thus, our present study aimed to evaluate the impact of using the thermally modified mineral adsorbent shungite and the dried seaweed meal *Fucus vesiculosus* in different doses in the diets of Brown Nick cross laying hens on their productivity indicators, digestibility of nutrients, nitrogen utilization, morphological blood profile, immunity status indicators, and egg quality. Our results showed that the inclusion of the abovementioned feed additives improves both productive performance and immunity status of Brown Nick cross laying hens as well as decreases the incidence of fatty liver occurrence.

**Abstract:**

The main purpose of this study was to assess the impact of using the thermally modified mineral adsorbent shungite (MAS) and the dried seaweed meal *Fucus vesiculosus* (DSM) with different doses in Brown Nick cross laying hens’ diet on their productivity, nutrient digestibility, morphological and blood profile, immunity status, and egg quality. A total of 261,720 hens were used in this experiment at the age of 63 weeks, and they were randomly divided into 5 groups (feeding program) with six repetitions of 8724 chickens in each. The first served (control) as a control group where laying hens were fed the basal diet that was used on the farm only; the second and the third groups represented MAS+ and MAS++, where they received the basal diet supplemented by 0.1% and 0.25% (or 1.0 kg/t and 2.5 kg/t of feed) of the mineral adsorbent shungite (MAS) which was provided in the feed in powder form (5 microns) and was added to the feed at the feed mill; the fourth and fifth groups represented DSM+ and DSM++, which received the basal diet provided with 0.1% and 0.25% (or 1.0 kg/t and 2.5 kg/t of feed) of dried seaweed meal of *F. vesiculosus* algae (DSM). The average egg weight over the entire period of the experiment revealed significant differences between the experimental groups and represented in the control group 65.20 vs. 66.88, 66.87 and 68.10 and 68.13 g in the MAS+ and MAS++, and DSM+ and DSM++ groups, respectively. Once the dried seaweed meal F. vesiculosus (DSM) was used, the crude protein increased significantly (*p* < 0.05) in egg yolk by 2.64 and 2.67%, carotenoids by 1.13 and 1.20 mg/g DM. The inclusion of both MAS and DSM feed additives revealed a significant decrease in the level of crude fat (lipids) in their liver when compared with the control group. The level of erythrocytes (RBCs) increased (*p* < 0.05) in the MAS+ and MAS++ and DSM+ and DSM++ groups when compared to the control group. Similarly, a significant increase was noted in hemoglobin when DSM was supplemented when compared to the control one. Moreover, the number of heterophils increased (*p* < 0.05) in groups of MAS and DSM when compared to the control group. The percentage of phagocytic activity increased significantly by 5.39, 6.90, and 7.18% in MAS++, DSM+, and DSM++, respectively, relative to the control group. On the other hand, the phagocytic number decreased (*p* < 0.05) by 1.15 and 1.12 conditional units in MAS+ and MAS++ and by 1.03 and 0.83 conditional units in DSM+ and DSM++ when compared to the control group, respectively. Consequently, the inclusion of thermally modified mineral adsorbent shungite and the dried seaweed meal *F. vesiculosus* with different doses in Brown Nick cross laying hen diets improves the egg weight and egg quality, crude protein, carotenoids and vitamin A in the egg mass, the utilization of lysine and methionine nutrients, hemoglobin content, immunity status, while decreases the incidence of fatty liver occurrence.

## 1. Introduction

Poultry farming is one of the intensively developing fields of agriculture [1]. The widespread use of poultry rearing is due, firstly, to the higher profitability of egg and meat production compared to other animal species; secondly, to the dietary and nutritional properties of the products; and thirdly, to the intensive growth, more rapid development of breeding in this industry, which determines the intensive development and increase in productivity of meat and egg production [2,3].

The use of antibiotics as growth stimulants [4], feed additives for the prevention of infectious diseases [5,6], and the use of other chemicals have become widespread, but a number of these drugs have a significant drawback—all these substances remain in the final products of poultry [7,8], which leads to a significant hazard to human health status.

The residual amount of antibiotics in poultry products can cause the adaptation of many pathogens, which contributes to the development and spread of antibiotic-resistant bacteria. Moreover, the increased risk of infection with antibiotic-resistant infections that can spread in an uncontrolled environment, and their use in feeding animals producing products for humans, can affect human health, economy, and national security [9,10].

Another important direction for increasing the productivity of poultry is the providing of new feed products suitable for saturating poultry diets with missing nutrients [11]. The use of algae can be a promising direction in this area [12,13,14]. The most developed in this respect is the use of unicellular algae in animal feeding (*Spirulina*, *Chlorella*, *Spirogyra*, etc.) [15]. Their widespread use in human and animal nutrition is due to lower costs for their production, ease of rearing, and rich chemical composition [16,17]. However, in the territory of Russia in the coastal regions, especially the northern seas, there is a high content of complex marine algae, especially the *Fucophyceae* [14]. Brown algae include the largest and most complexly organized marine protists. They include 1500 species, which are grouped into 265 genera, which include *Laminaria*, *Sargassum*, *Cystoseira*, *F. vesiculosus* [18]. Many authors have noted that brown algae are distinguished by good nutrition, are characterized by a balanced content of amino acids, including essential acids, contain a complex of biologically active substances, chelated compounds, and have a positive effect on the viability, productivity, and reproductive qualities of various animal species [19,20]. 

However, these studies were mainly carried out on algae growing in warmer climates, mainly in the coastal countries of the Japan [21], the EU [17], the USA, and the United Kingdom [22]. In Russia, the main place of growth of *Fucus* algae is the coastal zone of the seas of the Arctic and Atlantic Oceans, which is due to their chemical composition and the level of biologically active substances [8,23]. 

One of the alternative products is the use of modern sorbents based on shungite rocks with particle sizes up to 1 mm, which are mined on an industrial scale at the Zazhoginskoye in the Republic of Karelia [24].

Consequently, the present research aimed to study the impact of using the thermally modified mineral adsorbent shungite (MAS) and the dried seaweed meal *F*. *vesiculosus* in different doses in the diets of Brown Nick cross laying hens on their productivity indicators, digestibility of nutrients, nitrogen utilization, morphological blood profile, immunity status indicators, and egg quality.

## 2. Materials and Methods

This animal study was reviewed and approved by the Ethics Committee of the Russian State Agrarian University—Moscow Timiryazev Agricultural Academy (protocol 2022-8 date 6 May 2022).

### 2.1. Experimental Design, Diets and Bird’s Management

All studies were carried out on the basis of a commercial poultry farm Limited Liability Company Agricultural Enterprise “Voskhod” (53°56′25.5″, 56°23′52.7″) the village of Rodina of the Gafuriysky district of the Republic of Bashkortostan, Russia. In total, 261,720 cross Brown Nick commercial female layer hens were used in the experiment at the age of 63 weeks, the live weight was 2.03 kg, the average egg-laying capacity of one laying hen was 367 eggs, with an average egg weight of 62.7 g. The experiment began at 63 weeks and ended at 100 weeks of egg laying (all studies were conducted in 2021–2022). Hens were randomly assigned to six separate poultry houses. Within each poultry house, there were 5 groups (feeding program) of laying hens with 8724 hens in each group and received the following rations: control (received the basal diet that was used on the farm without additions); MAS+ (received the basic diet that was used on the farm supplemented by 0.1 (or 1.0 kg/t of feed) of the mineral shungite (MAS)); MAS++ (received the basic diet that was used on the farm supplemented by 0.25% (or 2.5 kg/t of feed) of the mineral shungite); DSM+ (received the basic diet that was used on the farm supplemented with 0.1% (or 1.0 kg/t of feed) of dried seaweed meal (DSM) of *Fucus* algae; and DSM++ (received the basic diet that was used on the farm supplemented with 0.25% (or 2.5 kg/t of feed) of dried seaweed meal of *Fucus* algae. The laying hens were fed the previous supplements with their compound feeds that corresponded to the commercial guidelines for the maintenance and feeding of the Brown Nick breed [25]. The composition and chemical nutritional analysis of the basal diet is presented in Table 1.

Laying hens of each group were kept in a one poultry house, which was composed of a single room with a cage poultry rearing system. The conditions of confinement in all five groups are identical and correspond to the technological parameters adopted for the maintenance of laying hens of the Brown Nick crossbred. Chickens were kept in an ecologically controlled room with free access to water and kept in 3-level cages with adjustable ventilation and lighting (lighting duration: 16 h). The eggs were collected and weighed at the same time every day. The technology of watering involves the use of nipple drinkers. The experimental birds were subjected to veterinary treatment according to the scheme of preventive measures adopted at the enterprise.

### 2.2. Diets and Analysis Nutrition

The hens of all groups were fed the same full-fledged compound feeds, which were balanced in nutrients, metabolic energy, and amino acid ratio and corresponded to the recommended nutrition characteristics of laying hens [25]. Feeding was free, and access to water was not restricted.

The studied additive was introduced into the composition of compound feeds as part of a premix by stepwise mixing. Formulas for complete feed for poultry were developed using the computer program Feed Optima (v. 2020.8.17251), taking into account the chemical composition of the components, which was determined at the Russian State Agrarian University—Moscow Timiryazev Agricultural Academy (Moscow, Russia).

### 2.3. Sample Collection and Chemical Analysis

The chemical composition was determined before the production of compound feed in accordance with the Association of Official Methods of Analytical Chemists [26,27].

Before the formulation of rations, each raw material was analyzed for estimating its nutritional value. The moisture content (%) in all samples was determined by drying the sample at 100–105 °C for 24 h (DM) (method AOAC 930.15). All components of the feed and feces were analyzed for the content of the main nutrients: DM (AOAC 934.01), EE (AOAC 920.39), and CP (AOAC 968.06). The determination of crude ash was carried out by the AOAC method (942.05) by burning egg mass samples at 500–550 °C for 6 h.

The analysis of the mineral composition (silicon, zinc, aluminum, iron, manganese, calcium, magnesium, sodium, potassium, sulfur, cobalt, and copper) of the adsorbent from shungite rock was determined according to ISO 6869:2000 on a double-beam atomic absorption spectrophotometer AA-7000 (Shimadzu, Kyoto, Japan). Phosphorus was determined using the Shimadzu AA-7000 atomic absorption spectrophotometer (Shimadzu Corporation, Tokyo, Japan) with a wavelength range ranging from 185 to 950 nm with a permissible relative measurement error of no more than 5% in the range of mass concentrations with KH_2_PO_4_ as State Standard Russian Federation No. 30615-99 [28]. Calcium was determined using an atomic absorption spectrophotometer according to State Standard Russian Federation No. 55573-2013 [29]. The content of the ether extract was determined by extraction with petroleum ether (AOAC method 920.39). The crude protein content (N × 6.25) (AOAC 976.05 method) was determined by the Kjeldahl method using the Basic Labtec Foss digester (Foss Electric LLC, Hollered, Denmark) and the KT 200 Kjeltec™ distillation unit (Foss Electric LLC, Hollered, Denmark).

### 2.4. Productivity and Quality Detection of Eggs of Cross Brown Nick Chickens

During the experiment, the weekly live weight, egg production, egg production rate, egg weight, and feed intake in each group were recorded, and the feed/egg ratio and daily feed intake (DFI) were calculated.

The following indicators were taken into account:

1. Live weight (g)—by control individual weighing of chickens (100 heads from each group) at the beginning and at the end of the experiment.
2. Safety of livestock (%)—by daily accounting of the case with clarification of its causes and calculation as a percentage of the initial livestock;
3. Egg production of a layer chickens placed indoors (JHEP)—was determined by dividing the number of eggs laid during the experiment by the number of chickens in the group at the beginning of the experiment;
4. The intensity of egg production (%)—by determining the ratio of the number of eggs laid during the experiment to the number of feeding days, expressed as a percentage;
5. Feed costs per 1000 pcs. eggs (kg)—by dividing the amount of feed consumed for the entire period of the experiment by 1000 eggs.
6. Feed costs per head per day (g)—by dividing the amount of feed consumed for the entire period of the experiment by the head of the bird.
7. Average egg weight (g) by weighing using electronic laboratory scales Mercury 122ACF-3000.05 (Mercury WP Tech Group Co., Ltd., Seoul, Republic of Korea).

At the age of 48 weeks, 35 eggs were randomly selected from each group for each treatment (3X) to evenly represent all repeats and were used to determine the qualitative parameters of the eggs as described by Attia et al. (2020) [30]. Before chemical analysis, the selected eggs were weighed on a scale with an accuracy of 0.01 g, then broken into a porcelain bowl, mixed with the shell, and dried at a temperature of 65 °C in a drying cabinet. The eggs were collected in accordance with the European Regulations (EC No. 1/2005 and EC No. 1099/2009) on the care of animals and their welfare. Sampling did not affect the well-being of hens, as it was carried out when the animals were not in the nests, which allowed avoiding contact with them.

The weight of laying eggs was calculated as the productivity of laying eggs (%) × daily egg weight (g). A sample weight of 5.0 g was placed in a round-bottomed flask with a capacity of 300 cm^3^, and 50 cm^3^ of ethyl alcohol, 100 mg of pyrogallol, and 3 g of granular sodium hydroxide (NaOH) were added. The contents of the flask were heated in a water bath with a reflux condenser for 40 min. After the hydrolysis was completed, the flask was quickly cooled under a stream of cold water and the contents were transferred to a separation funnel. The flask was washed with 30 cm^3^ of distilled water and drained into a separation funnel. A sign of complete saponification was the transparency of the mixture when water was added. Vitamin A was extracted with diethyl ether using successively 40, 25, and 25 cm^3^ of diethyl ether.

The combined essential extract was transferred to a separating funnel and washed from an alkali to a neutral medium, while consuming 500–700 cm^3^ of distilled water. Anhydrous sodium sulfate was added to the washed extract and left for 30–40 min in the dark at room temperature. Then, the ether was distilled under vacuum on a rotary evaporator until a dry residue was obtained, which was dissolved in 25 cm^3^ of ethyl alcohol.

The resulting alcohol solution of vitamin A standard was analyzed on a Shimadzu Prominence-i LC2030C Plus liquid chromatograph (Shimadzu Corporation, Tokyo, Japan) with a diode-matrix detector, with a Nucleodur C 18 Gravity column (150 × 3.8 mm in size with a sorbent whose grain size is 5 microns). The temperature of the column was 30 °C. A mixture of acetonitrile and methanol (80:20) was used as the mobile phase. Elution was carried out in a gradient mode. The feed rate of the mobile phase was 0.6 cm^3^/min, and the volume of the injected sample was 20 µL. The signal was recorded at a wavelength of 324 nm. The retention time of vitamin A is 1.7 min. The quantitative determination of vitamin A was carried out by the method of absolute calibration by the areas of chromatographic peaks. The linear detection range was in the range of 0.2–4.0 mg/cm^3^.

The content of carotenoids was determined according to the National Standard of the Russian Federation 54058-2010 (Functional foods and foods for special dietary uses. Method for determination of carotenoids) by extraction of carotenoids from a sample previously obtained by treating the sample with solutions of Karreza I and Karreza II. Subsequent purification of the isolated preparation with petroleum ether and spectrophotometric determination of mass concentration was determined by spectrophotometric measurement in fractions obtained during chromatographic separation of the extract on the spectrophotometer Unico 2100 (United Products & Instruments, Inc., Dayton, NJ, USA).

The determination of vitamin B_2_ in yolk and protein was carried out in accordance with Interstate Standard Russian Federation EN 14152-2013 using high-performance liquid chromatography at Shimadzu Prominence-i LC2030C Plus (Shimadzu Corporation, Tokyo, Japan) [31]. The fat content in eggs was determined according to State Standard of Russian Federation 31469-2012 by hydrolysis of the sample with hydrochloric acid, extraction of the released fat with diethyl and petroleum ethers, and evaporation of ether and weighing of the dry residue [32]. Ash content in eggs was determined according to State Standard of Russian Federation 54607.10-2017 by burning with a gradual increase in temperature for 5–6 h to 550 °C until a gray-white color is achieved [33]. The protein analysis was performed in the same way as the feed in Section 2.3.

### 2.5. Digestibility of Nutrients and the Use of Nitrogen in the Diet

The digestibility and use of nutrients of the diets were measured based on the results of balance experiments conducted according to the methodology of the Institute of Poultry Breeding of the Russian Academy of Sciences (2015) [34]. For this experiment, five chickens were selected from each group, homogeneous in their live weights and reflecting the average value for the group. The birds were kept in special cages with a mesh floor, under which a pull-out tray for collecting litter was installed.

The fecal matter was collected for each bird, cleaned of feed and feathers, and then weighed, dried in an oven with forced air supply at a temperature of 65 °C for several hours, then the samples were finally crushed and placed in dark glass jars with a screw-down lid until the analyses were carried out. Preservation and analysis of samples were carried out similarly as described in our earlier work [4].

Apparent digestibility coefficients (*DC*, %) of nutrients in the diet was evaluated using Equation (1):

(1) $$DC=\frac{intake\;nutrient-excreted\;in\;feces}{intake\;nutrient}\times 100\%$$

The analysis of nutrient absorption was carried out according to the standard methodology of the Institute of Poultry Breeding of the Russian Academy of Sciences (2015) [34]. Analysis of the content of essential amino acids (lysine, methionine) was carried on the amino acid analyzer Sicam S 433 (Sykam GmbH, Erding, Germany), and nitrogen and calcium are the same as in Section 2.3.

### 2.6. Collection and Analysis of Blood and Hepatic Samples

To assess the blood parameters and the direction of metabolism in the bird’s body, we took blood from the 35 laying hens to study the biochemical and morphological parameters. To do this, 68 weeks after the start of the experiment, 35 hens from each group were randomly selected (in 2 repetitions from each group). Whole blood samples were collected using vacuum tubes with K2 EDTA (Guangzhou Improve Medical Instruments Co., Ltd., Huangyan, China). Morphological parameters were determined in the blood, erythrocytes, platelets, leukocytes, basophils, eosinophils, heterophiles, lymphocytes, monocytes, and hemoglobin were analyzed using an automatic analyzer Abacus Junior 5 (Vet) (DIATRON MI ZRT., Budapest, Hungary) using AVANTOR control materials. Coloring: by May-Grunwald (GeMAStandart, St. Petersburg, Russia), microscopy using Meiji Techno, Nikon.

### 2.7. Statistical Analysis

The results are expressed as the means ± standard errors. Data were statistically analyzed using the statistical analysis program SPSS, 2017 [35]. Tukey’s multiple comparison tests (post hoc test) were used to compare the means at *p* < 0.05 of the treated groups [32]. One-way ANOVAs followed by Tukey’s multiple comparison tests (post-hock test) were used to check the significance and compare the experimental groups, according to the following statistical model:

X_ij_ = μ + A_i_ + E_ij_

where:

X_ij_ = An individual observation;

μ = Overall mean;

A_i_ = Effect of ith treatment.

E_ij_ = Random error.

## 3. Results

### 3.1. Chemical Composition of Feed Additives Applied

The analysis of the mineral content of thermally modified shungite was carried out before the start of the study and the obtained results are illustrated in Table 2.

According to our research, the mineral rock shungite contains total carbon of 34.8% by weight and mineral compounds (in the form of oxides, % by weight): Si (54.89), Al (3.67), Fe (2.43), Mn (<0.02), Ca (0.19), Mg (1.07), Na (<0.3), K (1.05), and P (0.06).

The nutritional value of dried seaweed meal of *F. vesiculosus* algae is presented in Table 3.

The analysis of the nutritional value of brown algae flour from the White Sea of the Republic of Karelia showed that the content of crude feed protein was 22.10%, crude fiber was 7.30%, fat was 12.90%, and the total amount of minerals was 19.30%, including the proportion of iodine of 0.10%.

### 3.2. Productive Performance Indicators and Feed Efficiency of Brown Nick Cross Laying Hens

The productivity indicators of Brown Nick cross laying hens at the age of 63 to 72 weeks are shown in Table 4, and the characteristics of egg quality in Table 5.

The control group (received the basal diet that was used on the farm without additions); MAS+ (received the basic diet that was used on the farm supplemented by 0.1 (or 1.0 kg/t of feed) of the mineral shungite (MAS)); MAS++ (received the basic diet that was used on the farm supplemented by 0.25% (or 2.5 kg/t of feed) of the mineral shungite); DSM+ (received the basic diet that was used on the farm supplemented with 0.1% (or 1.0 kg/t of feed) of dried seaweed meal (DSM) of *Fucus* algae); and DSM++ (received the basic diet that was used on the farm supplemented with 0.25% (or 2.5 kg/t of feed) of dried seaweed meal of *Fucus* algae).

The intensity of egg laying by a laying hen from an average live chicken at the age of 72 weeks during the experiment period was in (247.9, 250.4, 253.4, and 254.3 vs. 244.2) in MAS+, MAS++, DSM+, and DSM++ when compared to the control group, respectively. At the same time, the safety of poultry in the five groups was relatively high and represented (99.36, 99.39, 99.04, 99.51, and 99.33%).

Despite the increase in feed costs per 1 head per day in the MAS+ and MAS++ groups by 1.4 and 1.5 g and in the DSM+ and DSM++ groups by 1.6 and 1.0 g compared to the control one when recalculating the amount of feed costs per 1000 laid eggs, the Brown Nick cross laying hens receiving MAS++ in the feed composition, exceeding the control group by 0.4 kg, while those groups that received DSM+ and DSM++ were lower than the control group (T1) by 2.2 and 4.7 g, respectively.

The average egg weight laid by layer hens over the entire period of the experiment revealed significant differences between the experimental groups and represented in the control group 65.20 vs. 66.88, 66.87 and 68.10 and 68.13 g in the MAS+, MAS++, DSM+, and DSM++ groups, respectively. Among category B eggs, a significantly higher number (5.23%) was occupied by eggs soiled in manure in the control group, to a lesser extent (5.14%) in the MAS+ and MAS++ groups.

**Figure 1:**
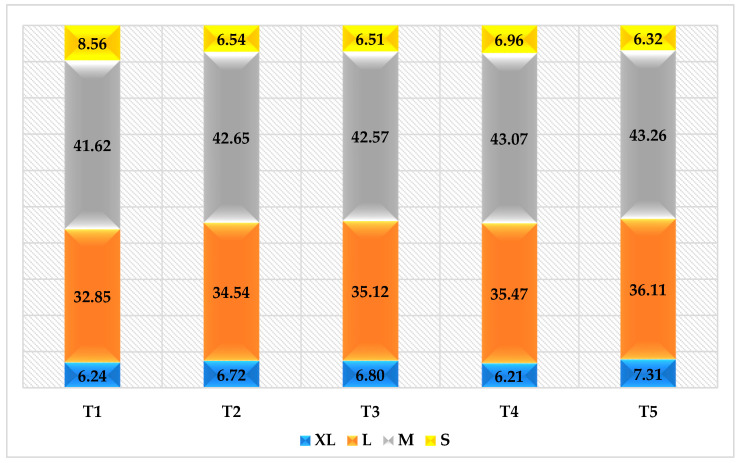
Impact of using the thermally modified mineral adsorbent shungite and the dried seaweed meal *Fucus vesiculosus* on the characteristics of eggs of laying hens of the of Brown Nick cross laying hens. S, M, L, and XL = small, medium, large, and extra-large size.

The results of eggs depending on their size and weight are shown in Figure 1.

Laying hens that consumed MAS and DSM as part of their diets had a higher proportion of XL and L category eggs than control poultry. Chicken eggs obtained from the control group had the highest proportion of size S (7.56%) and a very small proportion of size XL (5.24%).

### 3.3. Digestibility of Feed Nutrients of Brown Nick Cross Laying Hens

Means ± standard errors for the digestibility coefficient of laying hens of Brown Nick cross laying hens are presented in Table 6.

The introduction of MAS+, MAS++, DSM+, and DSM++ feed additives did not significantly affect the digestibility of crude protein, ether extracts, and crude fiber in all chicken groups. According to the utilization of nutrients during the present experiment, a significant increase in the use of lysine and methionine in the diet was recorded in the group receiving DSM compared to the control group.

### 3.4. Chemical Analysis of Egg Mass and Hepatic of Brown Nick Cross Laying Hens

The effect of the introduction of MAS and DSM feed additives in the diet of laying hens on the chemical composition of eggs during the experimental period is presented in Table 7 and liver in Table 8.

It was noted that when DSM was introduced into the feed of laying hens, it affected the accumulation of protein in the egg mass. Thus, when DSM was introduced into the feed in addition to phosphorus, the crude protein increased significantly (*p* < 0.05) by 2.64 and 2.67%, carotenoids by 1.13 and 1.20 mkg/g, and vitamin A in the yolk by 0.86 and 1.33 mkg/g in (DSM+ and DSM++ groups vs. control (control), respectively.

It was noted that the inclusion of MAS and DSM feed additives in the diets of Brown Nick cross laying hens revealed a significant decrease in the level of crude fat (lipids) in their liver when compared with the control laying hens and it represented (35.02, 35.12, 35.10, and 35.08 vs. 37.14%), respectively.

### 3.5. Morphological Blood Parameters and Immunity Status of Brown Nick Cross Laying Hens

Results of morphological parameters of blood of Brown Nick cross laying hens are illustrated in Table 9.

Conferring to the hematological analysis, it was recorded a significant increase in the level of erythrocytes (RBCs) in the MAS+, MAS++, DSM+, and DSM++ groups when compared to the control group by 0.59, 0.47, 0.5, and 0.55 × 10^12^/L relative to control, respectively. Similarly, an increase (*p* < 0.05) in hemoglobin was noted when DSM was introduced into the diet of Brown Nick cross laying hens by 5.60 and 6.04 g/L when compared to the control one. The number of heterophiles significantly increased in groups of Brown Nick cross laying hens received MAS and DSM in their diets when compared to the control group.

Indicators of immunity status of Brown Nick cross laying hens during the experiment period are given in Table 10.

The introduction of MAS and DSM feed additives into the diets of Brown Nick cross laying hens had a positive significant effect on phagocytic activity. The percentage of phagocytic activity increased significantly by 5.39, 6.90, and 7.18% in MAS++, DSM+, and DSM++, respectively, relative to the control group. On the other hand, the phagocytic number decreased (*p* < 0.05) by 1.15 and 1.12 in MAS+ and MAS++, and by 1.03 and 0.83 conventional units in DSM+ and DSM++ when compared to the control laying hens, respectively.

## 4. Discussion

### 4.1. Performance Indicators and Feed Efficiency of Brown Nick Cross Laying Hens

The quality of eggs of laying hens is an important indicator of poultry production due to the fact that they are considered as a good and economically justified source of high-grade protein in the diet of the human population, especially with limited financial resources [36,37].

The safety of livestock during the entire research period was relatively high and amounted to 99.04–99.51%, respectively. Probably, the introduction of brown algae into the compound feed had a positive effect on the state of immune function, which had a positive effect on the safety of livestock and resistance to pathogenic microorganism MAS. However, in our studies, the effect on the microbiota was not studied, but we have a plan to investigate this during further studies. When MAS and DSM were introduced into the feed, feed costs increased by 1 unit, however, the cost of producing 1000 eggs decreased. On the contrary, the intensity of egg production remained at a constant level in the CON and MAS groups throughout the experiment.

The results of our research refute the statements of Borzouei et al. (2020) [38], who mentioned that feeding brown algae powder (*Ascophyllum nodosum*) from the 31st to the 72nd week to Lohmann white and Lohmann Brown crosses did not significantly affect the change in production characteristics. In the studies of commercial feed additives from brown algae (*Ascophyllum nodosum*) [39], which were administered in an amount of 0.25% and 0.5% of the feed weight in Lohmann Lite chickens at the age of 70 weeks, significant changes in the weight of eggs and shells (0.25% dosage) were shown, at the same time a higher dosage (0.5%) algae did not have a significant impact on these indicators.

Our studies are consistent with the results of the work of Stupart (2019) [39], where an increase in egg weight was observed. Probably, the greatest importance in the obtained result was assigned to the mineral composition and the content of biologically active substances of dry seaweed flour, which were better absorbed in the poultry of the experimental group compared to other feeds. At the same time, the addition of an additive from mineral raw materials of the Republic of Karelia did not significantly affect the weight of the egg, however, there are differences in this group in the content of category B eggs (unsuitable eggs); in the experimental groups their number was significantly lower. One of the factors reducing the marketability of eggs was a soft shell, or its absence, among all groups; a smaller number of such eggs were in the group receiving MAS.

### 4.2. Digestibility of Feed Nutrients of Brown Nick Cross Laying Hens

The digestibility and assimilation of nutrients in laying hens and broilers is one of the important elements that ensure increased productivity and safety of poultry [40].

In our studies, non-significant differences in the digestibility of crude protein, essential extract, and crude fiber were found. According to our research, the use of dried algae flour contributed to the better use of lysine, which is probably due to the better digestibility of this amino acid from algae and stimulation of the digestive processes of poultry.

According to Černa M. (2011) [41] and Morris et al. (2020) [20], in seaweed, essential amino acids (BCAAs) account for almost half of the total amount, and their protein profile is close to that of egg protein. It is likely that the best assimilation of amino acids (lysine and methionine) is because the content of these amino acids in algae is quite high and because of the available form is better absorbed by poultry.

Thus, according to studies by Stokvis et al. (2022), it was reported [42] that their nutrients and biologically active algae compounds modulate the immune system, increase the efficiency of nutrient use and growth rates, improve the quality and stability of products, and prevent outbreaks of diseases in poultry [17].

### 4.3. Chemical Analysis of Egg Mass and Liver of Brown Nick Cross Laying Hens

Improving the egg quality, taste, and nutritional properties are of top importance in the field of production and management of poultry products [43,44]. In our research, it was found that the protein content increased in the group that received dry flour from brown algae of the White Sea. This phenomenon is probably due to the full-fledged composition of algae, which is confirmed by the similar results obtained in the works [45,46,47,48]. In studies by Carrillo et al. (2008) [45], where when brown algae *Macrocystis pyrifera* was included in the feed in an amount of 10% in the diet of 35-week-old leghorn chickens, it effectively increased the content of omega-3 fatty acids in the egg, protein height, and yolk color. In another study, Al-Harthi et al. (2012) [46] studied the effect of brown algae *Sargassum dentifebium* in the amount of 3% and 6% in the diet of laying hens and found it significantly reduced the level of cholesterol in the yolk and triglycerides, and also improved the level of carotene in eggs.

### 4.4. Morphological Blood Parameters and Immunty Status of Brown Nick Cross Laying Hens

Morphological indicators and nonspecific immunity in the blood of commercial poultry are markers of health in the production of eggs and poultry meat in the system of industrial poultry farming [8,49]. A significant increase in the level of erythrocytes in the DSM group by 0.5 × 10^12^/L relative to CON was recorded. Similarly, an increase in hemoglobin and the number of heterophiles were noted when DSM was introduced into the diet (*p* < 0.05). Moreover, the introduction of DSM to laying hens in the feed had a positive effect on phagocytic activity while the phagocytic number decreased in the supplemented groups MAS and DSM (*p* < 0.05).

According to a review by Mlambo et al. (2022) [50], bioactive compounds have been reported to modulate the immune system, improve nutrient utilization efficiency and growth rates, improve product quality and stability, and prevent disease outbreaks in poultry. At the same time, the rapid growth in demand for seaweed as an alternative to antibacterial and anti-inflammatory drugs of chemical synthesis is due to the use and search for natural bioactive products [51].

The probability of increasing the indicators of immune function by the content of biologically active components (fucoidan, alginic acid, tocopherol, bioflavonoids, selenium, etc.) [20], determines detoxification, anticarcinogenic, and immunomodulatory effects [47].

Rendering to studies conducted on poultry on the use of blue–green algae conducted by Benedetta et al. (2004) [52], a positive effect of algae was reported on the antioxidant and immune system of the body due to stimulation of the microbiota of the animal body. At the same time, it was noted in [20,47,53,54,55] that the use of alginate, fucoidan, and laminarin—the main polysaccharides of obtained and brown algae, have a protective effect on the intestinal tract, regulating the intestinal microbiota, increasing the expression of tight junction proteins (TJP), inhibiting the expression of inflammatory factors, and suppressing oxidative stress to restore damage to the intestinal barrier.

## 5. Conclusions

We can conclude that the inclusion of thermally modified mineral shungite and the dried seaweed meal *F. vesiculosus* with different doses in the diets of Brown Nick cross laying hen diets improves the egg quality through increasing the average egg weight laid, protein content in eggs and vitamin A in the yolk, the utilization of lysine and methionine nutrients, hemoglobin content, immunity status, while decreasing the incidence of fatty liver occurrence. Based on the present results the recommended dose for use is 2.5 kg/t of feed for MAS and 2.5 kg/t of feed for DSM. Moreover, further information is required for investigating the effect of using MAS and DSM together in one group either synergistic or antagonistic.

## Figures and Tables

**Table 1:** The composition of the basal diet of Brown Nick cross laying hens.

Ingredients	Content (%)	Parameter	Nutritional Value (%)
Wheat	40.9	Metabolic energy (kcal/100 g)	250
Barley	8.5	Crude protein	16.41
Peas	9.0	Crude fiber	5.69
Oats (contain few hulls and therefore, less fiber content)	9.0	Crude fat	3.30
Soybean meal	4.0	Linoleic acid	1.78
Sunflower meal	15.5	Lysine	0.79
Sunflower oil	1.6	Methionine	0.41
Lysine hydrochloride 98%	0.21	Methionine + cystine	0.66
DL-methionine 98.5%	0.15	Tryptophan	0.19
Sodium chloride	0.20	Calcium	3.46
Monocalciumphosphate	1.0	Assimilable phosphorus	0.37
Limestone powder	8.8	Sodium	0.15
Sodium sulfate anhydrous	0.14	Chlorine	0.21
Vitamin-trace mineral premixes	1.0	DEB (mgEq/100 g)	15.32

DEB (mgEq/100 g) = dietary electrolyte balance (mg equivalent in 100 g of diet). Note: Vitamin and mineral premixes (per kg of diet): vitamin A—1200 thousand ME/kg; vitamin D—300 _Tыc_. ME/kg; vitamin E—2000 mg/kg; vitamin K—200 mg/kg; vitamin B_1_—200 mg/kg; vitamin B_2_—600 mg/kg; vitamin B_3_ (niacin)—2000 mg/kg; vitamin B_4_—50,000 mg/kg; vitamin B_5_ (pantothenic acid)—2000 mg/kg; vitamin B_6_—400 mg/kg; vitamin B_12_—2.5 mg/kg; vitamin Bc—100 mg/kg; vitamin C—5000 mg/kg; vitamin H (biotin)—15 mg/kg; Fe—2500 mg/kg; Cu—250 mg/kg; Zn—7000 mg/kg; Mn—10,000 mg/kg; Co—100 mg/kg; I—70 mg/kg; Se—20 mg/kg.

**Table 2:** Mineral composition of mineral shungite.

Indicators	Content, % by Weight	Chemical Element	Content, % by Weight
SiO_2_	54.890	Silicon (Si)	23.070
Al_2_O_3_	3.670	Aluminum (Al)	6.000
Fe_2_O_3_	2.430	Iron (Fe)	3.500
MgO	1.070	Magnesium (Mg)	1.800
CaO	0.190	Calcium (Ca)	1.200
SO_3_ (sulfates)	0.7200–0.7862	Sulfur (S)	1.200
K_2_O	1.050	Potassium (K)	1.010
Na_2_O	<0.300	Sodium (Na)	0.250
MnO	<0.020	Manganese (Mn)	0.022
P_2_O_5_	0.060	Phosphorus (P)	0.080
Zn	0.008	Zinc (Zn)	0.008
Carbon (total)	34.800	Carbon (total)	34.800
Copper (Cu)	0.0058	Copper (Cu)	0.0058

**Table 3:** Nutritional value of dried seaweed meal of *F. vesiculosus*.

Indicators	Content, %
Dry matter	91.52
Crude fat	12.90
Crude ash	19.57
Crude fiber	7.30
Organic matter	84.22
Crude protein	22.10
Mass fraction of iodine	0.10
Mass fraction of alginates	29.80
Mass fraction of fucoidan	3.24
Mass fraction of polyphenols	0.80
Nitrogen-free extractive substances	29.65

**Table 4:** Impact of using the thermally modified mineral adsorbent shungite and the dried seaweed meal *Fucus vesiculosus* on productivity indicators of Brown Nick cross laying hens.

Parameters	0	MAS+	MAS++	DSM+	DSM++
Survivability (safety) of birds (%)	99.36	99.39	99.04	99.51	99.33
Egg production for the initial laying hen (eggs)	244.2	247.9	250.4	253.4	254.3
Intensity of egg production (%)	80.82	80.92	81.04	81.31	81.52
Feed consumption per head per day (g)	117.0	118.4	118.5	118.6	118.0
Feed consumption per 1000 eggs (kg)	146.6	146.6	147.0	143.8	141.9

0MAS/DSM = no mineral adsorbent shungite (MAS) and dried sweat meal (DSM) of *Fucus* algae. MAS+ = (1.0 kg/t of feed) MAS; MAS++ = (2.5 kg/t of feed) MAS; DSM+ = (1.0 kg/t of feed) DSM; DSM++ = (2.5 kg/t of feed) DSM.

**Table 5:** Impact of using the thermally modified mineral adsorbent shungite and the dried seaweed meal *Fucus vesiculosus* on category and quality defects of egg shells Brown Nick cross laying hens.

Parameters	0	MAS+	MAS++	DSM+	DSM++	*p*-Value
Mean egg weight (g)	65.20 ± 0.90 ^b^	66.88 ± 0.68 ^ab^	66.87 ± 0.50 ^ab^	68.10 ± 0.57 ^a^	68.13 ± 0.71 ^a^	0.034
Egg categories by quality (category B, %):	10.61 ± 0.50 ^a^	8.77 ± 0.48 ^ab^	8.74 ± 0.39 ^ab^	8.14 ± 0.23 ^b^	8.13 ± 0.55 ^b^	0.016
Soft and weak shelled eggs (%)	0.15 ± 0.02	0.13 ± 0.02	0.14 ± 0.03	0.14 ± 0.04	0.13 ± 0.03	0.995
Broken and minor cracks on the shell (%)	5.23 ± 0.33	5.14 ± 0.38	5.21 ± 0.30	5.03 ± 0.24	5.03 ± 0.39	0.987
Chicken eggs soiled in manure (%)	5.23 ± 0.36 ^a^	3.50 ± 0.43 ^b^	3.39 ± 0.34 ^b^	2.97 ± 0.29 ^b^	2.97 ± 0.24 ^b^	0.004
Egg categories by quality (category A, %)	89.39 ± 1.77	91.23 ± 0.89	91.26 ± 0.99	91.86 ± 0.70	91.87 ± 0.76	0.053

0 = no mineral adsorbent shungite (MAS) and dried sweat meal (DSM) of *Fucus* algae. MAS+ = (1.0 kg/t of feed) MAS; MAS++ = (2.5 kg/t of feed) MAS; DSM+ = (1.0 kg/t of feed) DSM; DSM++ = (2.5 kg/t of feed) DSM; Category A = eggs are characterized by a normal clean, intact shell and cuticle. category B = eggs do not meet the specified requirements and can only be used by the food industry or for non-food purposes. Values are expressed as means ± standard error. Means denoted within the same row with different superscripts are significant (*p* < 0.05).

**Table 6:** Impact of using the thermally modified mineral adsorbent shungite and the dried seaweed meal *Fucus vesiculosus* on digestibility of feed nutrients of Brown Nick cross laying hens.

Parameters	0	MAS+	MAS++	DSM+	DSM++	*p*-Value
Crude protein	89.7 ± 1.20	89.5 ± 1.06	90.2 ± 1.24	90.7 ± 0.97	90.8 ± 0.78	0.467
Essential extract	73.8 ± 0.93	73.9 ± 1.41	74.0 ± 1.54	74.3 ± 1.21	74.4 ± 1.11	0.691
Crude fiber	19.6 ± 1.19	20.2 ± 1.12	20.1 ± 1.47	21.6 ± 1.08	21.2 ± 0.98	0.339
Nitrogen	46.7 ± 1.02	46.8 ± 1.10	47.6 ± 0.87	48.0 ± 0.96	48.1 ± 1.02	0.364
Lysine	80.8 ± 0.28 ^b^	80.5 ± 0.18 ^b^	80.9 ± 0.17 ^b^	81.9 ± 0.22 ^a^	81.8 ± 0.31 ^a^	0.047
Methionine	80.2 ± 0.43 ^b^	80.0 ± 0.52 ^b^	80.3 ± 0.39 ^b^	80.9 ± 0.46 ^b^	81.2 ± 0.27 ^a^	0.009
Calcium	46.1 ± 1.28	46.1 ± 1.21	46.7 ± 1.02	46.0 ± 1.51	46.5 ± 1.74	0.725

0MAS/DSM = no mineral adsorbent shungite (MAS) and dried sweat meal (DSM) of *Fucus* algae. MAS+ = (1.0 kg/t of feed) MAS; MAS++ = (2.5 kg/t of feed) MAS; DSM+ = (1.0 kg/t of feed) DSM; DSM++ = (2.5 kg/t of feed) DSM; Values are expressed as means ± standard error. Means denoted within the same row with different superscripts are significant (*p* < 0.05).

**Table 7:** Impact of using the thermally modified mineral adsorbent shungite and the dried seaweed meal *Fucus vesiculosus* on chemical analysis of egg mass of Brown Nick cross laying hens.

Parameters	0	MAS+	MAS++	DSM+	DSM++	*p*-Value
Crude protein, % in DM	47.08 ± 0.76 ^b^	48.00 ± 0.75 ^ab^	48.07 ± 0.48 ^ab^	49.72 ± 0.36 ^a^	49.75 ± 0.87 ^a^	0.024
Crude ash, %	3.31 ± 0.17	3.35 ± 0.18	3.41 ± 0.25	3.37 ± 0.21	3.52 ± 0.34	0.582
Calcium, %	0.26 ± 0.02	0.27 ± 0.03	0.27 ± 0.05	0.26 ± 0.03	0.28 ± 0.03	0.562
Phosphorus, %	0.74 ± 0.03 ^b^	0.75 ± 0.02 ^b^	0.75 ± 0.02 ^b^	0.76 ± 0.02 ^b^	0.81 ± 0.02 ^a^	0.023
Carotenoids, mkg/g	11.66 ± 0.20 ^b^	11.88 ± 0.29 ^ab^	11.74 ± 0.32 ^b^	12.79 ± 0.37 ^a^	12.86 ± 0.15 ^a^	0.001
Vitamin A in the yolk, mkg/g	13.20 ± 0.24 ^b^	13.30 ± 0.22 ^ab^	13.25 ± 0.27 ^b^	14.06 ± 0.20 ^a^	14.53 ± 0.18 ^a^	0.003
B_2_ in yolk, mkg/g	4.80 ± 0.29	4.77 ± 0.30	4.78 ± 0.25	4.92 ± 0.40	5.03 ± 0.34	0.608
B_2_ in protein, mkg/g	4.67 ± 0.29	4.79 ± 0.31	4.78 ± 0.30	4.88 ± 0.32	4.97 ± 0.24	0.779

0MAS/DSM = no mineral adsorbent shungite (MAS) and dried sweat meal (DSM) of *Fucus* algae. MAS+ = (1.0 kg/t of feed) MAS; MAS++ = (2.5 kg/t of feed) MAS; DSM+ = (1.0 kg/t of feed) DSM; DSM++ = (2.5 kg/t of feed) DSM; Values are expressed as means ± standard error. Means denoted within the same row with different superscripts are significant (*p* < 0.05).

**Table 8:** Impact of using the thermally modified mineral adsorbent shungite and the dried seaweed meal Fucus vesiculosus on chemical composition of liver of Brown Nick cross laying hens.

Parameters	0	MAS+	MAS++	DSM+	DSM++	*p*-Value
Crude protein, % in DM	45.42 ± 2.13	45.91 ± 3.54	46.13 ± 2.68	46.02 ± 1.35	45.94 ± 1.14	0.904
Crude ash, %	3.31 ± 0.10	3.29 ± 0.14	3.28 ± 0.17	3.32 ± 0.19	3.34 ± 0.14	0.899
Crude fat, %	37.14 ± 0.26 ^a^	35.02 ± 0.58 ^b^	35.12 ± 0.25 ^b^	35.10 ± 0.54 ^b^	35.08 ± 0.42 ^b^	0.002

0MAS/DSM = no mineral adsorbent shungite (MAS) and dried sweat meal (DSM) of *Fucus* algae. MAS+ = (1.0 kg/t of feed) MAS; MAS++ = (2.5 kg/t of feed) MAS; DSM+ = (1.0 kg/t of feed) DSM; DSM++ = (2.5 kg/t of feed) DSM; Values are expressed as means ± standard error. Means denoted within the same row with different superscripts are significant (*p* < 0.05).

**Table 9:** Impact of using the thermally modified mineral adsorbent shungite and the dried seaweed meal *Fucus vesiculosus* on morphological blood parameters of hen of Brown Nick cross laying hens.

Parameters	0	MAS+	MAS++	DSM+	DSM++	*p*-Value
RBC, 10^12^/L	2.60 ± 0.19 ^b^	3.19 ± 0.36 ^ab^	3.07 ± 0.40 ^ab^	3.10 ± 0.22 ^ab^	3.15 ± 0.18 ^a^	0.040
WBC, 10^9^/L	35.85 ± 3.93	30.26 ± 4.13	34.21 ± 3.47	31.22 ± 2.78	35.96 ± 2.45	0.681
Thrombocytes, 10^9^/L	71.84 ± 11.34	65.71 ± 10.84	67.64 ± 10.45	62.67 ± 22.58	72.12 ± 24.14	0.768
Hb, g/L	80.00 ± 1.48 ^b^	83.00 ± 8.85 ^ab^	82.9 ± 1.24 ^ab^	85.60 ± 2.18 ^a^	86.4 ± 2.43 ^a^	0.028
ESR, mm/h	1.60 ± 0.49	1.20 ± 0.40	1.34 ± 0.45	1.20 ± 0.40	1.41 ± 0.14	0.711
MCH, pg	1.68 ± 0.21 ^b^	1.79 ± 0.44 ^a^	1.84 ± 0.26 ^a^	1.99 ± 0.11 ^a^	2.03 ± 0.37 ^a^	0.021
Basophils, %	1.0 ± 0.45	1.2 ± 0.20	1.1 ± 0.42	0.8 ± 0.37	1.2 ± 0.34	0.686
Monocytes, %	0.4 ± 0.24	0.8 ± 0.37	0.7 ± 0.24	0.8 ± 0.20	0.9 ± 0.19	0.205
Heterophils, %	6.61 ± 0.51 ^c^	12.04 ± 0.84 ^b^	11.64 ± 0.54 ^b^	18.83 ± 1.46 ^a^	19.11 ± 1.24 ^a^	0.001

0MAS/DSM = no mineral adsorbent shungite (MAS) and dried sweat meal (DSM) of *Fucus* algae. MAS+ = (1.0 kg/t of feed) MAS; MAS++ = (2.5 kg/t of feed) MAS; DSM+ = (1.0 kg/t of feed) DSM; DSM++ = (2.5 kg/t of feed) DSM; Values are expressed as means ± standard error. Means denoted within the same row with different superscripts are significant (*p* < 0.05). ESR—erythrocyte sedimentation rate; MCH—mean level of hemoglobin in the erythrocyte; RBC—red blood cells; WBC—white blood cells; Hb—hemoglobin.

**Table 10:** Impact of using the thermally modified mineral adsorbent shungite and the dried seaweed meal *Fucus vesiculosus* on indicators of immunity status of Brown Nick cross laying hens.

Parameters	0	MAS+	MAS++	DSM+	DSM++	*p*-Value
Phagocytic activity, %	21.59 ± 0.95 ^b^	26.88 ± 2.65 ^ab^	26.98 ± 2.12 ^a^	28.49 ± 1.52 ^a^	28.77 ± 1.24 ^a^	0.002
Phagocytic index, %	10.64 ± 0.58 ^ab^	9.93 ± 0.72 ^ab^	9.87 ± 0.64 ^b^	11.01 ± 0.46 ^ab^	11.68 ± 0.54 ^a^	0.040
Phagocytic number, convent. units	4.95 ± 0.28 ^b^	3.80 ± 0.35 ^a^	3.83 ± 0.45 ^a^	3.92 ± 0.28 ^a^	4.12 ± 0.19 ^a^	0.013

0MAS/DSM = no mineral adsorbent shungite (MAS) and dried sweat meal (DSM) of *Fucus* algae. MAS+ = (1.0 kg/t of feed) MAS; MAS++ = (2.5 kg/t of feed) MAS; DSM+ = (1.0 kg/t of feed) DSM; DSM++ = (2.5 kg/t of feed) DSM; Values are expressed as means ± standard error. Means denoted within the same row with different superscripts are significant (*p* < 0.05).

## Data Availability

The raw data supporting the conclusions of this article will be made available by the authors without undue reservations. The data presented in this study are available upon request from the corresponding author.
